# The influence of the Gilgel-Gibe hydroelectric dam in Ethiopia on caregivers' knowledge, perceptions and health-seeking behaviour towards childhood malaria

**DOI:** 10.1186/1475-2875-9-47

**Published:** 2010-02-11

**Authors:** Delenasaw Yewhalaw, Wondwossen Kassahun, Kifle Woldemichael, Kora Tushune, Morankar Sudaker, Daniel Kaba, Luc Duchateau, Wim Van Bortel, Niko Speybroeck

**Affiliations:** 1 Department of Biology, Jimma University, Jimma, Ethiopia; 2 Department of Statistics, Jimma University, Jimma, Ethiopia; 3 Department of Epidemiology, Jimma University, Jimma, Ethiopia; 4 Department of Health Planning and Health Management, Jimma University, Jimma, Ethiopia; 5 Department of Health Education, Jimma University, Jimma, Ethiopia; 6 Jimma Zone Health Bureau, Ministry of Health, Jimma, Ethiopia; 7 Department of Physiology and Biometrics, University of Ghent, Ghent, Belgium; 8 Department of Parasitology, Institute of Tropical Medicine, Antwerp, Belgium; 9 Department of Animal Health, Institute of Tropical Medicine, Antwerp, Belgium; 10 Public Health School, Université Catholique de Louvain, Brussels, Belgium

## Abstract

**Background:**

Malaria remains the most important public health problem in tropical and subtropical areas. Mothers' or caregivers' ability to recognize childhood malaria-related morbidity is crucial as knowledge, attitudes and health seeking behavior of caregivers towards childhood malaria could influence response to signs of the disease.

**Methods:**

A total of 1,003 caregivers in 'at-risk' villages in close proximity to the Gilgel-Gibe hydroelectric dam in south-western Ethiopia, and 953 caregivers in 'control' villages further away from the dam were surveyed using structured questionnaires to assess their knowledge, perceptions and health seeking behaviour about childhood malaria.

**Results:**

Malaria (*busa*) was ranked as the most serious health problem. Caregivers perceived childhood malaria as a preventable ('at-risk' 96%, 'control' 86%) and treatable ('at-risk' 98% and 'control' 96%) disease. Most caregivers correctly associated the typical clinical manifestations with malaria attacks. The use of insecticide-treated nets (ITNs) was mentioned as a personal protective measure, whereas the role of indoor residual spraying (IRS) in malaria prevention and control was under-recognized. Most of the caregivers would prefer to seek treatment in health-care services in the event of malaria and reported the use of recommended anti-malarials.

**Conclusion:**

Health education to improve knowledge, perceptions and health-seeking behaviour related to malaria is equally important for caregivers in 'at risk' villages and caregivers in 'control' villages as minimal differences seen between both groups. Concluding, there may be a need of more than one generation after the introduction of the dam before differences can be noticed. Secondly, differences in prevalence between 'control' and 'at-risk' villages may not be sufficient to influence knowledge and behaviour.

## Background

Malaria remains a serious public health problem, causing 1.2 million deaths [1] and 300 to 660 million clinical cases in tropical and subtropical areas each year [2]. More than 90% of the lethal cases occur in children under five years of age in Africa [3]. In Ethiopia, malaria is one of the most important health problems with nearly 52 million (68%) of the populations being at risk to malaria infection [4]. It is the leading cause of morbidity and mortality, accounting for 10-20% of hospital admissions and 10-40% of severe cases in children under 5 years and 13-26% of all inpatient admissions in various health facilities with corresponding proportional mortality rates of 13-35% [5].

*Plasmodium falciparum *and *Plasmodium vivax *are predominant parasite species responsible for 60% and 40% of the infections, respectively. *Plasmodium malariae *and *Plasmodium ovale *account for less than 1% of the cases [6]. Malaria transmission is seasonal and unstable. The prevention of malaria in Ethiopia has relied mainly on early diagnosis and treatment of infection and reduction of human-vector contact by indoor residual spraying (IRS) and large-scale distribution of insecticide-treated nets (ITNs) and long-lasting insecticidal nets (LLINs) [7].

Mothers' or caregivers' ability to recognize childhood malaria-related morbidity is crucial as about 80-90% of malaria cases are treated at home in Africa [8,9] and several studies indicate that knowledge, attitudes and practices (KAP) of caregivers towards childhood malaria could influence response to signs of the disease [10,11]. Moreover, lack of knowledge and misconceptions of caregivers about the transmission and treatment of malaria may also affect malaria control interventions in general and jeopardize effective malaria treatment and home malaria management in particular.

Therefore, this study was conducted to report on caregivers' knowledge and perceptions about the causation, transmission, prevention, treatment and treatment-seeking behaviour of childhood malaria. The aim of this study was to generate baseline information on the KAP of caregivers' about malaria in the context of the potential impact of the Gilgel-Gibe dam on malaria incidence and transmission as diseases such as malaria may change in transmission dynamics or increase in incidence due to large dam reservoirs [12]. The study may also help to implement effective intervention strategies to prevent and control malaria in communities living in close proximity to the dam.

## Methods

### Study area

The study area is located 260 km south-west of the capital, Addis Ababa in Oromia Regional State, south-western Ethiopia near Gilgel-Gibe hydroelectric dam, the biggest dam in Ethiopia, which started operating in 2004. The study area lies between latitudes 7°42'50"N and 07°53'50"N and between longitudes 37°11'22"E and 37°20'36"E, at an altitude of 1,672-1,864 m above sea level. The area has a sub-humid, warm to hot climate, receives between 1,300 and 1,800 mm of rain annually and has a mean annual temperature of 19°C. The rainfall pattern of the area is similar to other parts of Ethiopia with the long rainy season starting in June and extending up to September, while the short rainy season begins in March and extends to April/May. The main socio-economic activities of the local communities are mixed farming involving the cultivation of staple crops (maize, teff and sorghum), combined with cattle and small stock-raising. The study villages are located in Sekoru, Tiro-Afeta, Omo-Nada and Kersa districts (*weredas*). Houses are traditional type constructed of mud and wood, the majority with thatched roofs and very few with corrugated iron sheets.

### Study design and data collection

A base line survey was conducted in 16 villages from May to July 2007. The study population comprised children less than 10 years of age who lived for at least six months in the selected villages. All villages surrounding the dam (within a 10 km radius) were classified into 'at-risk' or 'control' according to their distance from the dam. Villages within 3 km from the dam were identified as test ('at-risk') villages and the remaining villages, 5-10 km from the dam were identified as 'controls'. A total of eight pairs of 'at-risk' and 'control' villages were selected so that they were as similar to each other as possible, except for the distance to the dam. Details of the procedure of the selection of study villages are described elsewhere [13]. The sample size was chosen such that the study would detect an 8% incidence difference of *P. falciparum *(15% in the 'at-risk' and 7% in the 'control' group) allowing for a dropout rate of 15%, with α = 0.05 and β = 0.20 using a 1:1 ratio. This resulted in a sample of 2082 children under 10 years of age (1041 in at 'at-risk and 1041 in 'control' villages). A complete list of all children younger than 10 years of age in the 16 villages was obtained and 130 children were randomly selected for each 'at-risk and 'control' villages. A multi-stage cluster design was employed, each village being a primary sampling unit (PSU) and children below 10 years of age as secondary sampling units (SSUs).

Information on knowledge, perceptions and health-seeking behaviour was gathered from the caregivers of those 2082 children. Data were collected by 12 community health extension workers (CHEWs) who were residents in the selected villages and well conversant (speak, read and write) of the local language (*Afaan Oromo*). They were hired and trained how to interview and adhere to the survey protocol. Each household (defined by members living together) was visited and numbered, and for each household member (resident in the village for greater than or equal to six months of the year) the name, age, and sex was recorded. Each house was assessed in terms of its structure, accessibility and proximity to the dam. The survey was conducted using a pre-tested semi-structured questionnaire having both closed and open-ended questions. The structured questionnaire was first developed in English and then translated into the local language (*Afaan Oromo*) and administered in the local language to the caregiver of each selected child less than 10 years of age in both 'at-risk' and 'control' villages. The questionnaire contained the following categories of questions: demographic characteristics, socio-economic factors, common human ailments in the area, childhood malaria related episodes in children in the preceding two weeks, and knowledge and perception questions related to malaria transmission, causations, signs, symptoms, burden and severity of the disease, treatment-seeking behaviour, local prevention and control practices. The questionnaire was piloted in two villages which were not selected for the study. A face-to-face interview schedule was arranged to collect relevant data from each caregiver during house-to-house visits. Interviews were conducted privately to maintain confidentiality and avoid family and peer-pressure.

### Data analysis

Data were checked for completeness and consistency and then data were double entered into the computer. The data analysis was based on Stata 11 (Stata Corp, College Station, TX, USA). Summary statistics were computed for the study variables in 'at-risk' and 'control' groups separately. Odds ratios (OR) with 95% confidence intervals (95% CIs) were computed to compare the two groups of caregivers using the χ^2^-test at a significance level of 5%. The clustering at village level was taken into account during the data analysis.

### Ethical consideration

Ethical approval of the study was obtained from Jimma University Research and Ethics Committee after study protocol review and the proposal was also reviewed and approved by the World Health Organization Ethical Review Committee (ERC). Permission from the community was sought before initiating the study by communicating the responsible zonal and district administrative offices through official letters from Jimma University. Similarly, community agreement and local oral consent was sought from village leaders through meetings with villagers. Individual informed oral and written consent were sought from each caregiver in the caregivers' local language, *Afaan Oromo*, for all literate caregivers. An independent literate witness from village leaders confirmed verbal consent for illiterate caregivers of the selected children. Confidentiality of case identity was maintained for all records.

## Results

### Socio-demographic characteristics of respondents

One thousand and three caregivers from 'at-risk' villages and 953 caregivers from 'control' villages were interviewed. Table 1 shows the socio-demographic and socio-economic profile of the study subjects. In both the 'at-risk' and 'control' villages, the majority of the caregivers were parents (either mother or father) of the study children (97.4% and 96.4%, respectively). Females constituted the majority of caregivers with 882 (87.9%) and 771 (80.9%) women in 'at-risk' and 'control' villages, respectively. The male to female ratio was 1:7.3 in 'at-risk' villages and 1:4.2 in 'control' villages. A total of 875 (87.2%) caregivers from at-risk and 787 (82.6%) from 'control' villages were above the age of 30 years. The median age was 36 years among caregivers of the 'at-risk' group and 35 years among 'controls'. The median household size was six in both groups of villages. The majority of caregivers in the two groups of villages were married (96.6 for 'at-risk' and 94.8% for 'control' villages), illiterate (80.9% for 'at-risk' and 85.2% for 'control' villages), and engaged in farming activity (96.2% 'at-risk' and 99.5% 'control'). Eight hundred and eighty-seven (88.4%) caregivers in 'at-risk' and 830 (87.1%) caregivers in 'control' villages lived in mud-walled and thatched roof traditional tukuls.

**Table 1:** Socio-demographic characteristics of caregivers of children less than 10 years of age for the 'at-risk' villages (n = 1003) and 'control' villages (953) in Gilgel-Gibe dam area, southwestern Ethiopia, 2007

Variable	Attribute	'At-risk' Count (%)	'Control' Count (%)
**Relationship with the child**	Mother/father	977 (97.4)	919 (96.4)
	Other relatives	26 (2.6)	30 (3.2)
	Non relatives	0	4 (0.4)
**Sex**	Female	882 (87.9)	771 (80.9)
	Male	121 (12.1)	182 (19.1)
**Age group (years)**	<30	128 (12.8)	166 (17.4)
	≥30	875 (87.2)	787 (82.6)
**Marital status**			
	Single	12 (1.2)	19 (2.0)
	Married	969 (96.6)	903 (94.8)
	Divorced	3 (0.3)	3 (0.3)
	Widowed	19 (1.9)	28 (2.9)
**Educational level**	Illiterate	811 (80.9)	812 (85.2)
	Read and write only	120 (12.0)	104 (10.9)
	Elementary	68 (6.8)	30 (3.1)
	Secondary education	2 (0.2)	5 (0.5)
	Above secondary education	2 (0.2)	2 (0.2)
**Occupation**	Farmer	965 (96.2)	948 (99.5)
	House wife	12 (1.2)	1 (0.1)
	Others	26 (2.6)	4 (0.4)
**Type of house (roof)**	Thatched	887 (88.4)	830 (87.1)
	Corrugated iron sheet	116 (11.6)	123 (12.9)

### Reported common childhood illnesses in the community by caregivers

**Figure 1:**
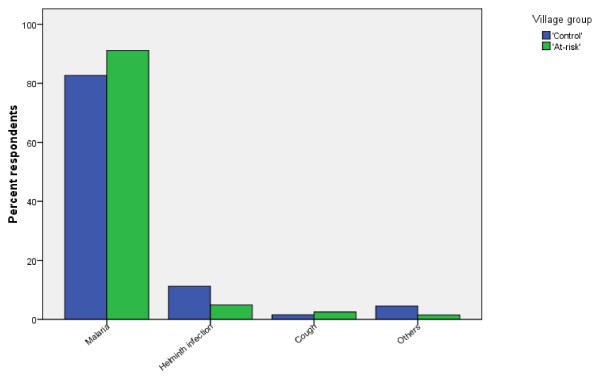
**Common childhood illnesses reported by caregivers of children less than 10 years of age in 'at-risk' villages and 'control' villages in Gilgel-Gibe dam area, south-western Ethiopia, 2007**.

Figure 1 shows the distribution of common childhood illnesses reported by caregivers in the study area. Nine hundred and fourteen (92.2%) and 788 (86.0%) caregivers mentioned *Busa *(the local name in *Afaan Oromo *for uncomplicated malaria) as the most common childhood illness and most frequent health concern in the 'at-risk' and 'control' villages, respectively. Other common childhood illnesses reported by the caregivers were helminthes infections (4.9% 'at-risk' and 11.7% 'control') and cough (2.5% 'at-risk' and 1.6% 'control'). Very few caregivers in each group reported that HIV/AIDS, tuberculosis and typhoid were common childhood illnesses.

### Reported malaria illness and case management of childhood malaria

The proportion of caregivers reporting a malaria episode in children two weeks preceding the study was higher in the 'at-risk' (OR = 1.49, 95%CI = 0.46-4.76) than in the 'control' villages though the difference was not statistically significant (p = 0.48) (Table 2). Seventy six percent and 85% of the caregivers from 'at-risk' and 'control' villages, respectively, reported that they sought treatment for children with malaria. With regard to the first action taken with their sick child, the following responses were provided by caregivers: taking to health services, treating with anti-malarial drugs and herbal preparations at home with no significant difference between the two groups of caregivers (p > 0.05). The majority of caregivers (82% for 'at-risk' and for 79% 'control' villages) reported that they used anti-malarial drugs to treat their sick child. With regard to the type of anti-malarial drug used, no important differences were found between the two groups of caregivers. Use of Coartem^® ^(artemether-lumefantrine), chloroquine and Fansidar^® ^(sulphadoxine-pyrmethamine)(SP) was reported by both groups of caregivers for treating their children suffering from malaria. Very few caregivers from the 'control' group also reported the use of quinine. Nearly 95% of the caregivers from each group indicated that their sick child completed the full course of anti-malarial drugs. In contrast, only 4% of caregivers from each group reported that their sick child did not take the full course of treatment. There were no significant differences in both groups of caregivers' responses when asked why their sick children had not sought treatment (p > 0.05). The mentioned reasons for the absence of treatment by both groups of caregivers were: lack of money, distance of health services and child considered not being seriously ill.

**Table 2:** Distribution of reported cases with a malaria episode and management of childhood malaria by caregivers in the preceding two weeks prior to the study period in Gilgel-Gibe dam area, southwestern Ethiopia, 2007

Variable	'At-risk'	'Control'	OR (95%CI)	p-value
**Children with a malaria episode in the past two weeks**				
('at-risk', n = 1003; 'control', n = 953)	21.8%	15.8%	1.49 (0.46-4.76)	0.483
**Children receiving treatment**				
**(**'at-risk', n = 159; 'control', n = 208)	75.5%	84.6%	0.56 (0.29-1.08)	0.079
**The first action taken**				
('at-risk', n = 120; 'control', n = 176)				
Took the child to health services	60.0%	68.8%		0.265
Treat with anti-malarials at home	35.8%	23.3%		
Treat with herbs at home	2.5%	6.8%		
Other	1.7%	1.1%		
**Use of anti-malarial remedies**				
('at-risk', n = 120; 'control', n = 179)	81.7%	79.3%	1.16 (0.29-4.65)	0.820
**Type of anti-malarial(s) given**				
('at-risk', n = 98; 'control', n = 142)				
Coartem^® ^(artemether-lumefantrine (AL))	56.1%	74.6%		0.392
Chloroquine (CQ)	34.7%	21.8%		
Fansidar^® ^(sulfadoxine-pyrimethamine (SP))	9.2%	2.1%		
Quinine (QN)	0.0%	1.4%		
**The child finished the dose**				
('at-risk', n = 142; 'control', n = 98))				
Yes	95.9%	95.1%	1.22 (0.13-11.73)	0.853
**Reasons for absence of treatment**				
('at-risk', n = 39; 'control', n = 32)				
Lack of money	76.9%	53.1%		0.114
Health facility very far	5.2%	28.1%		
Child not seriously ill	15.4%	18.8%		
Others	2.6%	0.0%		

### Recognition of the main signs and symptoms of uncomplicated childhood malaria

**Figure 2:**
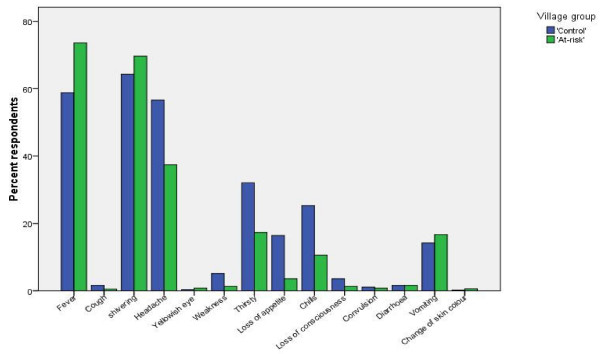
**Perceived signs and symptoms of uncomplicated childhood malaria by caregivers of children less than 10 years of age in 'at-risk' and 'control' villages in Gilgel-Gibe dam area, south-western Ethiopia, 2007**.

The caregivers' perceptions about the important signs and symptoms of malaria are shown in Figure 2. Most caregivers correctly associated the typical clinical manifestations with malaria attacks. Fever (hot body) was the most common (738 cases, 73.6%) symptom mentioned by the caregivers from the 'at-risk' group followed by shivering (613 cases, 64.3%), headache (375 cases, 37.4%), thirst (174 cases, 17.3%) and vomiting or nausea (168 cases, 16.7%). Caregivers from the 'control' group, however, mentioned shivering as the most common symptom (613 cases, 64.3%), followed by fever (560 cases, 58.8%), headache (539 cases, 56.6%), thirst (306 cases, 32.1%), chills (241 cases, 25.3%), loss of appetite (156 cases, 16.4%) and vomiting or nausea (135 cases, 14.2%). Loss of consciousness, convulsion and weakness were least mentioned by both groups of caregivers as symptoms or clinical manifestations of uncomplicated malaria.

### Cause and mode of transmission of childhood malaria as reported by caregivers

Reported responses of caregivers about the causes and transmission of childhood malaria are summarized in Table 3. Although statistically insignificant (OR = 2.05, 95% CI = 0.56, 7.57), caregivers from 'at-risk' villages recognized mosquito bites more often as a cause of malaria than caregivers from 'control' villages. Incorrect causes of malaria by both groups of caregivers were: exposure to sun, eating contaminated food, bad spirits, dirty and stagnant water, too much work and God's punishment. The majority of caregivers in each group of villages reported that malaria is transmitted through mosquito bites though there was a (non-significantly) lower level of knowledge among caregivers in the 'at-risk' (77%) than the 'control' group (83%) (OR = 0.64, 95% CI = 0.21, 1.93). Nearly 8% of caregivers responded that they did not know the route of malaria transmission (11% 'at-risk' and 4% 'control'). September to November was frequently mentioned as the main malaria transmission season (64% 'at-risk' and 50% 'control'). When asked about the usual biting time of mosquitoes, the majority of caregivers (76% 'at-risk' and 79% 'control') believed mosquitoes bite humans at night with no significant difference in the reported knowledge between the two groups of caregivers (p = 0.37). However, some caregivers (6% 'at-risk' and 4% 'control') reported that they did not know the usual biting time of mosquitoes. Stagnant water was the most frequently recognized breeding site of mosquitoes by both groups of caregivers (89% 'at-risk' and 78% 'control').

**Table 3:** Knowledge on causes and modes of transmission of childhood malaria reported by caregivers of children less than 10 years of age around Gilgel-Gibe hydroelectric Dam, southwestern Ethiopia, 2007

Attribute*	'At-risk'	'Control'	OR (95%CI)	p-value
**Cause of malaria** ('at-risk', n = 1003; 'Control', n = 953)				
Mosquito bite	59.1%	41.3%	2.05 (0.56, 7.57)	0.256
Exposed to the sun	51.3%	39.0%	1.65 (0.52, 5.25)	0.371
Eating contaminated food	13.5%	9.0%	1.57 (0.56, 4.38)	0.362
Bad spirit	3.3%	1.7%	1.99 (0.23, 17.28)	0.499
Dirty and stagnant water	2.9%	7.9%	0.35 (0.07, 1.64)	0.153
Too much work	1.6%	2.7%	0.58 (0.21, 1.60)	0.263
God punishment	1.1%	4.6%	0.23 (0.04, 1.23)	0.060
Body contact with patients	0.4%	7.9%	1.26 (0.20, 7.79)	0.791
Don't know	12.9%	4.3%	3.32 (0.48, 23.1)	0.187
**Modes of malaria transmission** ('at-risk; n = 984; 'control', n = 941)				
Mosquito bite	76.7%	83.7%	0.64 (0.21, 1.93)	0.400
Body contact with patients	10.9%	7.0%	1.62 (0.39, 6.75)	0.481
Via respiratory route	4.6%	8.5%	0.52 (0.11, 2.49)	0.377
Sharing utensils with patients	5.6%	2.4%	2.36 (0.44, 12.74)	0.281
Breast milk	8.2%	4.3%	2.02 (0.39, 10.38)	0.366
Don't know	11.4%	3.8%	3.23 (0.71, 14.70)	0.105
**Main transmission season** ('at-risk', n = 1003; 'control', n = 953)				
September to November	63.8%	49.5%		0.518
December to February	5.4%	5.9%		
March to April	8.4%	8.3%		
June to August	15.8%	24.9%		
Always	6.7%	11.2%		
**Usual mosquito biting time** ('at-risk', n = 755; 'control', n = 788)				
Day	4.8%	6.2%		0.379
Night	76.0%	78.6%		
Any time	13.0%	11.2%		
Don't know	6.2%	3.9%		
**Common mosquito breeding sites **('at-risk', n = 759; 'control', n = 784)				
Stagnant water	88.8%	90.4%		0.354
Running water	0.7%	1.8%		
Others	0.7%	2.8%		
Don't know	9.9%	4.9%		

* In all cases, the reference category is "No"

### Knowledge of malaria preventive methods, treatment and treatment-seeking behaviour of caregivers

The majority (91%) of caregivers believed that childhood malaria is a preventable disease (Table 4). However, the perception on preventability of childhood malaria was significantly higher (p = 0.03) in the 'at-risk' than in the 'control' group. One hundred thirty two (13.9%) and 39 (3.9%) caregivers from 'control' and 'at-risk' group, respectively, failed to recognize the preventability of childhood malaria.

**Table 4:** Knowledge and perception of caregivers on preventability, local prevention and control practices of childhood malaria for 'at-risk' and 'control' villages in Gilgel-Gibe dam area, south-western Ethiopia, 2007

Variable	Category	'At-risk' (%)^a^	'Control' (%)^a^	OR (95%CI)	p-value
**Preventability** ('at-risk', n = 1003; 'control', n = 953)	Yes	96.1%	86.1%	3.97 (1.04, 15.13)	0.033*
**Preventive methods**	Mosquito nets	73.1%	81.1%	0.63 (0.21, 1.98)	0.404
('at-risk', n = 965;	Repellant/Smoking	32.7%	12.9%	3.28(1.07,10.09)	0.035*
'control', n = 921)	Anti-malarial drugs	26.2%	8.0%	4.07 (1.06, 15.68)	0.034*
	Eliminating Breeding sites	17.6%%	4.6%	4.4 (1.25, 15.54)	0.017 *
	House spraying	16.5%	4.3%	4.41 (0.75, 25.81)	0.074
	Herbal preparation	1.9%	0.7%	2.73 (0.21, 36.37)	0.403
**Treatability** ('at-risk', n = 1003; 'control', n = 953)	Yes	97.8%	96.1%	1.80 (0.62, 5.24)	0.253
**Treatment-seeking behaviour**	Public health services	71.5%	65.1%	1.34 (0.37, 4.88)	0.632
('at-risk', n = 981;	Private health services	33.8%	20.4%	1.99 (0.53, 7.51)	0.281
'control', n = 916)	Self treat with herbs at home	1.8%	6.9%	0.25 (0.05, 1.32)	0.077
	Self treat with modern Medicine at home	1.4%	6.8%	0.19 (0.06, 0.63)	0.005
	Traditional healer	0.3%	0.5%	0.56 (0.08, 3.79)	0.521

* Significant at the 0.05 level

^a ^Multiple responses possible

The use of mosquito nets was the most frequently mentioned malaria preventive method and we found no difference in knowledge on mosquito nets as a preventive method between the two groups of caregivers. However, caregivers from the 'at-risk' group showed significantly higher perception of the usefulness of repellents (p = 0.034), anti-malarial drugs (p = 0.033) and elimination of breeding sites (p = 0.017) than caregivers from the 'control' group as methods of malaria prevention. Other less frequently reported preventive methods were indoor residual spraying (IRS) (16.5% 'at-risk' and 4.3% 'control') and herbal preparation (1.9% 'at-risk' and 0.7% 'control').

Approximately 97% of the caregivers in each group recognized childhood malaria as a treatable disease. With respect to treatment-seeking behaviour, government clinics were reported to be the first choice for treatment (71.5% 'at-risk' and 65.1% 'control') for a suspect child of having malaria, followed by private clinics (33.8% 'at-risk' and 20.4% 'control'), home treatment with herbal preparations (1.8% 'at-risk' and 6.9% 'control'), home treatment with anti-malarial drugs (1.4% 'at-risk' and 6.8% 'control') and taking the child to traditional healers (0.3% 'at-risk' and 0.5% 'control').

## Discussion

In this study, most caregivers were mothers, which is similar to the findings in other studies conducted in Tanzania and Uganda showing that 89% and 92% caregivers of children, respectively, were mothers [10,14].

Malaria was reported to be the most common childhood illness and the most serious health concern of caregivers in both groups of villages. Soil-transmitted helminth infection and HIV were also reported by caregivers as common childhood illnesses in the study area which is a matter of concern as the co-morbidity of soil-transmitted helminth infections with *P. falciparum *could result in severe anaemia in children [15,16] and malaria co-infection with HIV was also reported to increase the risk of severe falciparum malaria and malaria-related deaths [17].

The reported childhood malaria illness was higher in 'at-risk' than 'control' villages and this was consistent with the findings of the previous malaria point prevalence study conducted in two groups of similar villages in the same study area showing higher malaria prevalence in at-risk villages [13].

Results of this study also showed that the majority of caregivers who had diagnosed their child and reported to have uncomplicated childhood malaria in the two weeks prior to this study took some sort of action. A worrisome finding was that 24% of the caregivers from the 'at-risk' group and nearly 15% of the 'control' did not do anything for their children with reported childhood malaria. Of the caregivers who reported first-line treatment, the majority in each group said that they took the child to a health facility in contrast to the findings of other studies in Africa showing that only few caregivers took their children with uncomplicated malaria to a health facility [18]. The findings also indicate that 36% of the caregivers in 'at-risk' and 23% in 'control' group reported that in the first-line treatment they had given anti-malarial drugs to a child they diagnosed as having malaria at home, which is higher than the 6.4% reported in another study from northern Ethiopia [19]. Rates of self- or home treatment in Africa ranged from as low as 19% in Guinea to as high as 94% in rural Ghana [20]. The practice of self-treatment at home observed in this study may be an advantage as a short delay between disease onset and effective treatment has been linked to a lower risk of death [21,22].

The findings of this study further showed that caregivers in both groups had good knowledge on medication as most reported the use of AL and CQ (the current first-line anti-malarial drugs) for the treatment of uncomplicated falciparum and vivax malaria, respectively. This knowledge on the use of anti-malarials could contribute to the early and adequate treatment of malaria in the programme of home treatment of malaria [23]. Although SP is no longer recommended as medication due to resistance [6], its use indicates that it may still be available at some private health-care providers, drug shops or illegal vendors. Therefore, there may be a need for reorientation, follow-up and monitoring of the implementing this of the national anti-malarial treatment policy change by the national malaria control programme (NMCP), as failure to implement may result in increased malaria transmission [24]. Several reasons were reported for the absence of malaria treatment, such as lack of money, the distance of health services and low severity of the disease. This is consistent with another study in Uganda [25] showing that factors associated with prompt presentation at a health facility included severity of illness, proximity to a health facility and knowledge of malarial prevention methods. The frequently mentioned signs and symptoms of childhood malaria were fever, shivering and headache as reported by Govere *et al *[26]. Fever and shivering were the most recognized symptoms of childhood malaria by the caregivers which is in agreement with findings in other studies [11,19]. The recognition of fever by the majority of caregivers as a symptom of uncomplicated malaria could be of importance [23] and early treatment also depends upon prompt recognition of symptoms and signs of malaria in the household mainly by women [27].

Very few caregivers reported convulsion as symptom of childhood malaria which may indicate recognition of some features of severe malaria, which is in contrast to findings of several studies in Africa in which the appearance of convulsions were recognized but associated to some form of 'supernatural' force necessitating the involvement of a 'traditional healer' [28-30]. The majority of caregivers (77% 'at-risk' and 84% 'control' group) recognized that malaria is transmitted by mosquito bites, which is higher than the 5% and 48% observed in similar studies conducted in central and northern Ethiopia, respectively [31,32]. Moreover, mosquito bites as a cause of malaria were mentioned by more than half of the caregivers (both groups combined). This may be attributed to the efficiency of the local health service extension programme (HSEP), launched in 2004 by the Government of Ethiopia and designed to achieve basic health care coverage in the country through the provision of staffed health posts (one for 5,000 people). These health posts also deal with malaria prevention and treatment [33]. Mwenesi *et al *[18] indicated that there were inter-country, urban/rural and district variation in the quality and magnitude of sources of care available in a population. Most caregivers associated mosquito bites with malaria transmission which is important to sustainably run control programmes based on the use of ITNs. Minja *et al *[34] reported that where awareness of mosquitoes as malaria vector is present, the use of ITNs could be as high as 52%. More than half of the caregivers cited that malaria transmission would occur from September to November mainly. This is similar to most malaria endemic areas in the country where malaria is seasonal and unstable and the peak malaria transmission occurs after the rainy season. Most caregivers replied that mosquitoes bite humans during night time which was also reported in another rural community of central Ethiopia [35]. Caregivers' knowledge on the usual biting time of mosquitoes and seasonality of malaria could contribute to health extension workers efforts to promote the acceptance and use of ITNs by the community [36].

Most caregivers were quite knowledgeable about the preventability and treatability of malaria in contrast to the findings in central Ethiopia showing that 77% of the mothers believed that malaria could not be prevented [31]. Moreover, in the current study most respondents recognized the role of mosquito nets in malaria prevention. This may be useful information when implementing strategies to scale-up the distribution and use of ITNs. The NMCP distributed 20 million ITNs between 2005 and 2007 targeting 40 million people at risk [37] and aims to reach 100% ITNs coverage by the year 2010 [5]. Respondents acknowledged the use of repellents, chemoprophylaxis, eliminating mosquito breeding sites, house spraying and herbal therapy as personal prevention methods against malaria. However, some caregivers (9% both groups combined) believed that prevention or control of childhood malaria is impossible. There may therefore be a need to implement health education interventions to promote and improve malaria prevention and control.

The IRS programme had been reported to be highly successful in reducing malaria transmission [38,39] and spatial targeted vector control using IRS and ITNs were also found to reduce malaria transmission [40]. In the present study, the longstanding use of IRS activity or operation by the NMCP consists primarily in DDT use and occasionally in malathion. IRS is the main technical element of malaria prevention and control strategic approach in Ethiopia [41]. It was observed that the caregivers in both groups of villages had a poor perception of IRS in contrast to another study in south-western Ethiopia showing that the majority of the community knew the reason for house spraying [42]. This may hamper malaria control programmes as there may be less compliance by caregivers with spraying programmes, e.g., when replastering and washing treated wall surfaces after spray or when disallowing spray men entering into the houses for reasons of inconvenience or because of the bad odour of the insecticide. *Anopheles arabiensis*, the primary vector of malaria in the study area, was reported to be strongly endophilic [43]. Effective IRS against malaria vectors depends on the resting behaviour of mosquitoes [44] and on the knowledge of the malaria vector and the purpose of IRS [45]. It may, therefore, be important to educate caregivers to improve their knowledge about IRS, its risks and benefits so as to make them comply with the spraying programme.

The two groups of caregivers reported that they would seek treatment first in health services (health centers, clinics, health posts and malaria control laboratories) in contrast to other studies conducted in Africa which showed that most caregivers would use self-medication at home as the first line of treatment in the event of uncomplicated malaria in their children [23,46-48]. The choice for public health services may be attributed to these services currently being the sole sources of free anti-malarials. Indeed the Government of Ethiopia started taking measures through the HSEP to improve the prevention and treatment of malaria, distributing AL, CQ and QN free of charge either after diagnosis using a rapid diagnostic test (RDTs) or through clinical diagnosis by health extension workers at health posts. Such health posts are not far from the patients' homes as the NMCP recommends that the community should have access to health posts within less than 1-hour walking [33]. McCombie [49] stated that access, severity of illness and cost are the major factors determining use of health-care facilities. Though the majority of caregivers in both groups reported the use of health-care facilities as the first choice for treatment for their children with uncomplicated childhood malaria, caregivers also reported that the first response to the recognition of uncomplicated malaria was home treatment with anti-malarial drugs. This may call for involving community health extension workers to educate caregivers about home-based malaria treatment, anti-malarial drugs and dosages by delivering consistently anti-malarials. A study conducted in northern Ethiopia by Kidane and Morrow [50] indicated that effective treatment with anti-malarials at home and village levels of children with febrile illnesses resulted in prompt treatment and reduced incidence. Prompt recognition of febrile illnesses among children and early treatment with appropriate anti-malarial drugs at home or village level in rural endemic areas has become an alternative to health facility services [51]. Apart from lack of knowledge, distance to a health facility or cost of anti-malarials, the choice of treatment-seeking at home as the first line by some caregivers in this study could be attributed to the local social context. In Ethiopia, mothers or caregivers have a major role in the well-being of the household and are responsible for household chores. They may prefer home treatment as they may not get time for health-seeking at health services and keep stock of anti-malarials on hand for self-medication at home [52]. The peak malaria transmission season also coincides with the period of harvesting which may be another reason for not approaching health-care services as the time constraint may influence the treatment choice [49]. Few caregivers reported that they would seek treatment from traditional healers unlike the findings of a study in Uganda in which no caregivers reported to seek care from traditional healers [14].

## Conclusion

This study revealed that malaria is perceived as the commonest illness and most severe health concern for both groups of caregivers. The majority of caregivers sought treatment in health-care services for their child with malaria followed by home treatment using anti-malarials. Most importantly, caregivers of both groups have knowledge about the signs and symptoms of uncomplicated malaria (e.g., only few linked convulsion with malaria). Health education on how to recognize severe malaria may be needed and caregivers should be aware that uncomplicated malaria could progress into severe malaria. Knowledge of caregivers on personal protection methods and treatability of malaria was proper. A wide variety of personal protective methods were practiced by caregivers but knowledge on IRS as a protective method was low. Since poor knowledge on the purpose and use of IRS may jeopardize vector control activities to reduce level of malaria transmission in the study area, the NMCP should promote and improve the understanding of the function of IRS. Some of the caregivers reported the use of SP for the treatment of uncomplicated malaria and this should be appropriately addressed through reorientation, follow-up and monitoring. The small differences between the caregivers from 'at risk' and 'control' villages may be partially attributed to higher exposure of the caregivers of the 'at-risk' villages to malaria or to higher malaria prevalence compared to the caregivers of the 'control' villages after the introduction of the dam as the two groups of villages to some extent were previously supposed to be ecological replica of each other with similar malaria endemicity. Tanner *et al *[27] indicated that exposure to malaria is clustered, implying a cluster of knowledge and perception of malaria. However, in general, health education to improve the knowledge, perceptions and health-seeking behaviour related to malaria is equally important under different malaria burden settings. Indeed, the indicators of knowledge, perceptions and health-seeking behaviour related to malaria used in this study show that differences between caregivers in 'at-risk' villages and caregivers in 'control' villages were minimal. With respect to this, two observations can be made. Firstly, there may be a need of more than one generation after the introduction of the dam before differences can be noticed. Secondly, the prevalence observed by Yewhalaw *et al *[13], 6% and 12% in 'control' and 'at-risk' villages, respectively, may not be sufficient to influence knowledge and behaviour.

## Competing interests

The authors declare that they have no competing interests.

## Authors' contributions

DY conceived and designed the study, was involved in coordination, supervision and drafted the manuscript; WK involved in the study design, statistical analysis, supervision, coordination, data entry and cleaning; KW participated in the study design and reviewed the manuscript; KT involved in coordination and reviewed the manuscript; MS participated in supervision and development of survey instruments; DK involved in coordination and supervision next to MS; LD involved in statistical analysis and reviewed the manuscript; WVB critically reviewed the manuscript; NS performed the statistical analysis, was involved in drafting and critically reviewed the manuscript. All authors read and approved the final manuscript.
